# Andean Flora as a Source of New Repellents against Insect Pests: Behavioral, Morphological and Electrophysiological Studies on *Sitophilus zeamais* (Coleoptera: Curculionidae)

**DOI:** 10.3390/insects10060171

**Published:** 2019-06-14

**Authors:** Roberto Romani, Stefano Bedini, Gianandrea Salerno, Roberta Ascrizzi, Guido Flamini, Maria Cristina Echeverria, Priscilla Farina, Barbara Conti

**Affiliations:** 1 Department of Agricultural, Food and Environmental Sciences, University of Perugia, 06121 Perugia, Italy; gianandrea.salerno@unipg.it; 2 Department of Agriculture, Food and Environment, University of Pisa, 56124 Pisa, Italy; stefano.bedini@unipi.it (S.B.); priscilla21@rocketmail.com (P.F.); barbara.conti@unipi.it (B.C.); 3 Department of Pharmacy, University of Pisa, 56122 Pisa, Italy; roberta.ascrizzi@for.unipi.it (R.A.); guido.flamini@farm.unipi.it (G.F.); 4 Facultad de Ingeniería en Ciencias Agropecuarias y Ambientales, Grupo de Investigaciones Ambientales y Biotecnológicas, Universidad Tecnica del Norte, Ibarra 100105, Ecuador; mc.echeverria@hotmail.com

**Keywords:** maize weevil, essential oils, bioactivity, sensilla, ultrastructure, electroantennography, EAG

## Abstract

*Sitophilus zeamais* (Motschulsky) is considered as one of the most destructive foodstuff pests. Due to their efficiency, low toxicity for mammalians and low environmental impact, plant-derived essential oils (EOs) are promising tools for pest control. In particular, the OEs extracted from Lamiaceae are considered among the most bioactive in terms of repellent and/or insecticidal effect. Here, we investigated the repellence of the EOs extracted from two aromatic plant species typical of the flora of the Ecuadorian Andes, *Clinopodium tomentosum* and *C. nubigeum*, against adults of *S. zeamais*. The behavioral assays carried out at concentrations ranging from 0.7 to 23.9 μL L^−1^ air in a two-way static olfactometer showed a significant repellent effect starting from the concentration of 8.4 mL L^−1^ air both for the EO of *C. nubigenum* and *C. tomentosum*. We also carried out a complete structural analysis of the antenna of *S. zeamais* using scanning (SEM) and transmission electron microscopy (TEM), in order to characterize the olfactory sensilla equipment. In this species, there is no sexual dimorphism also as regards to the antennal morphology and the sensilla type and distribution. We identified six type of sensilla, among which at least three types (Sensillum Trichoideum 1, Sensillum Trichoideum 2 and Grooved Peg Sensillum) can be considered as olfactory. Electroantennography (EAG) recordings carried out on *S. zeamais* revealed a positive dose-response to both EOs, without differences between the two sexes.

## Highlights

- Sustainable control of stored product insect pests is receiving increasing attention, essential oils could represent an effective, environmentally-safe control method.
- Aromatic plants from megadiverse countries could be a source of new effective repellents or insecticides to be considered in IPM strategies.
- In this study we investigated the repellent effect of EOs from two Lamiaceae species of Ecuador Andean flora belonging to the *Clinopdium* genus against the maize weevil.
- The EOs where chemically characterized, then used for behavioral assays and EAG experiments. We also carried out a study on the antennal sensory structures of the maize weevil.
- EOs revealed to be perceived trough the antennal olfactory sensilla and showed a clear repellent activity towards the weevil.

## 1. Introduction

*Sitophilus zeamais* (Motschulsky) (Coleoptera: Curculionidae), the maize weevil, is one of the main pests of stored grain, causing either quantitative (in terms of loss of stored products), and qualitative (in terms of reduced quality of stored grains) damages in most part of the world [1,2]. Differently, from the closely related species, *S. granarius* L., but analogously to the rice weevil *S. oryzae* L., the maize weevil is able to fly, and can reach actively the grain storage facilities to initiate the infestation [3,4,5]. For this species, several control methods have been developed, from the use of pesticides (according to different formulations, including fumigations) to physical control methods (controlled atmosphere, low temperatures) [6,7,8,9]. As part of strategies aiming to achieve a sustainable control of this pest according to integrated pest management criteria, several organic substances were tested, including essential oils [10,11,12].

Essential oils (EOs) are natural extracts composed of a wide variety of volatile compounds, occurring as secondary metabolism products in several plant groups. EOs can be produced in different amounts by several plant organs, ranging from leaves, stems, seeds and flowers, from which they can be extracted using different methods [13]. EOs can be efficiently used to control insect pests because of their repellent effect, as well as their toxicity shown against different insect groups [14,15,16,17,18]. A large variety of EOs extracted from different plants, mainly belonging to the Lamiaceae family [19,20], were tested for their efficacy against *Sitophilus* spp. EOs from *Hyptis suaveolens* L. (Lamiaceae) and *Humulus lupulus* L. (Cannabaceae) exhibited a repellent activity against *S. granarius* [21,22], whilst EOs from *Ocimum tenuiflorum* L. (Lamiaceae) also exerted fumigant and toxic effects towards *S. oryzae* [23].

In this paper, we investigated the activity of the EOs extracted from two species of Lamiaceae belonging to the genus *Clinopodium*, *C. nubigenum* (Kunth) Kuntze and *C. tomentosum* (Kunth) Govaerts. The genus *Clinopodium* includes more than 140 species distributed worldwide. Several species of the genus *Clinopodium* are used as natural remedies in traditional medicine in different regions of the world [24]. This applies to *C. tomentosum* and *C. nubigenum* as well, which are commonly known in Ecuador as “Santa Maria” and “tipo de cerro”, respectively, and used in the traditional medicine as remedies against cold, flu, stomach-ache and other disorders [25]. EOs from collected plants of *C. nubigenum* and *C. tomentosum* were analyzed by gas chromatography (GC) and by gas chromatography/electron impact mass spectroscopy (GC/EIMS). To evaluate the effect of *Clinopodium* spp. EOs extracts, dual choice behavioural assays were performed. We also investigated the antennal morphology of *S. zeamais* to describe in detail the antennal sensilla, particularly as regards the olfactory sensilla. *Sitophilus* spp. was already investigated using mainly scanning electron microscopy (SEM) techniques [26,27,28], but a comprehensive study of the antennal structures in terms of fine structure and distribution is still lacking. We finally tested the antennal sensitivity to different concentrations of *C. nubigenum* and *C. tomentosum* EOs on *S. zeamais* males and females, using the EAG (electroantennography) method.

## 2. Materials and Methods

### 2.1. Essential Oils Chemical Analyses

Essential oils were extracted by hydrodistillation in a clevenger-type apparatus for two hours from the flowering aerial parts of plants collected in March 2017 on the mountains nearby the Hacienda Zuleta (Imbabura, Ecuador) collected with the Ecuadorian Environmental authorization No. 006-2017-IC-FAU-FLO-DPAI/MAE. The obtained EOs have been diluted to 0.5% in HPLC grade *n*-hexane prior to GC–MS injection. The GC/EI-MS analyses were performed with a Varian CP-3800 apparatus equipped with a DB-5 capillary column (30 m × 0.25 mm i.d., film thickness 0.25 μm) and a Varian Saturn 2000 ion-trap mass detector. The oven temperature was programmed rising from 60 °C to 240 °C at 3 °C/min; injector temperature, 220 °C; transfer-line temperature, 240 °C; carrier gas, He (1 mL/min); injection volume: 1 μL. The acquisition parameters were as follows: Full scan; scan range: 35–300 *m*/*z*; scan time: 1.0 Section; threshold: 1 count. The identification of the constituents was based on the comparison of their retention times (t_R_) with those of pure reference samples and their linear retention indices (LRIs) determined relatively to the t_R_ of a series of *n*-alkanes. The mass spectra were compared with those listed in the commercial libraries NIST 14 and ADAMS and in a homemade mass-spectral library, built up from pure substances and components of known oils, and MS literature data [29,30,31,32,33,34].

### 2.2. Insect Cultures and Rearing Conditions

*S. zeamais* population was reared under laboratory conditions (24 °C, 45–65% R.H., and in the dark) at the Department of Agriculture, Food and Environment of the University of Pisa. Plastic boxes (20 × 27 × 11 cm) containing corn and wheat, covered by a nylon net allowing air exchange, were used for the rearing. The bioassays were conducted on insects homogeneous in age, obtained by removing the adults present in the rearing boxes by sieving the grain and collecting the newly emerged insects (0–3 days old) the following day.

### 2.3. Behavioural Assays

The responses (attractiveness or repellence) of *S. zeamais* to the *C. nubigenum* and *C. tomentosum* EOs were evaluated by a still-air arena. The arena (15 × 15 × 1 cm), made of polymethylmethacrylate, consisted of three circular chambers (4 cm ∅). Two lateral chambers were connected to a central one (release chamber) by linear paths (2 cm in length, 1 cm in width), forming a 90° angle. The top of the arena was covered by means of a removable glass panel. In each replicate, a square of filter paper (0.5 × 0.5 mm) was treated with 3 μL of 0.3, 0.6, 1.25, 2.5, 3.5, 5.0 and 10.0% EO hexane solution (corresponding to 0.7, 1.4, 3.0, 6.0, 8.4, 12.0, and 23.9 μL/L air). After hexane evaporation, the treated filter paper was placed in one of the lateral chambers (cue chamber). A square of filter paper (0.5 × 0.5 mm) treated with 3 μL of hexane only was placed after hexane evaporation in the other lateral chamber (control chamber). At the beginning of the test, an adult was gently transferred to the floor of the release chamber and observed for 6 min. The insect was considered to have made a valid choice when it entered in one of the two lateral chambers after at least 20 s and remained in the chamber for at least 30 s. Insects that did not make a choice within 6 min after being released were disregarded. For each replicate we recorded (a) the latency time (i.e., the time between the insect release and the entering in one of the chambers), (b) the choice made and (c) the permanence time (i.e., the time spent in the chosen chamber). At each test, the arena was rotated clockwise 90° to avoid positional effects, the cue sources were renewed, and a new insect was placed in the arena. After three consecutive tests, the arena and the glass lid were washed for about 30 s with hexane, then with warm water at 35–40 °C and a mild soap for about 5 min, rinsed with hot water for about 30 s and finally, rinsed with distilled water at room temperature. For each EO concentration, the test was replicated until 30 unsexed adults made a valid choice. All behavioral assays were carried out over a period of several weeks to account for any daily variability. For each replicate, each insect was replaced by a new one of the same age (2–4 days old). All experiments were performed between 8:30 a.m. and 2:30 p.m. and conducted in a room uniformly lit with daylight fluorescent tubes (Philips 30 W/33). Light intensity was approximately 1000 lux (estimated over the 300–1100 nm waveband using a LI-1800 spectroradiometer LI-COR Inc., Lincoln, NE, USA equipped with a remote cosine receptor). Room temperature was set at 25 ± 1 °C and the relative humidity at 50 ± 5%.

### 2.4. Electron Microscopy Observation

Scanning electron microscopy (SEM). Ten adult individuals of each sex were used for the observations. Insects were anaesthetized by exposure to cold temperatures (−18 °C) for 120 s, then they were immediately soaked in 60% alcohol. The antennae of each individual were dissected from the head capsule as a whole; in a few cases, the last antennal segment was isolated from the rest of the antenna to achieve better view of the apical part of the antenna. Specimens were dehydrated in a series of graded ethanol, from 60% to 99%, 15 min for each step. After dehydration, 99% ethanol was substituted with pure HMDS (Hexamethyldisilazane, Merck^®^ KGaA, Darmstadt, Germany) and the specimens were allowed to dry under a hood, at room conditions; this step was repeated twice. Up to 5 samples were mounted on aluminium stubs, taking care to place them with different orientations in order to obtain a clear view of the ventral, dorsal and lateral sides of the antennomeres. Mounted specimens were gold-sputtered using a “Balzers Union^®^ SCD 040” unit (Balzers, Vaduz, Liechtenstein). The observations were carried out using a Philips^®^ XL 30 (Thermo Fischer Scientific, Hillsboro, OR, USA), SE detector, operating at 7–10 KV, WD 9–10 mm.

Transmission electron microscopy (TEM). Ten individuals of both sexes were anesthetized by exposure to cold temperatures (−18 °C) for 60 sec, then immediately immersed in a solution of glutaraldehyde and paraformaldehyde 2.5% in 0.1 M cacodylate buffer +5% sucrose, pH 7.2–7.3. Each antenna was detached from the head capsule, the last flagellomere was removed to help fixative penetration, and left at 4 °C for 24 h. Then, the specimens were washed in 0.1 M cacodylate buffer +5% sucrose, pH 7.2–7.3 for ten minutes, repeated twice. Afterwards, the specimens were post fixed in 1% OsO_4_ (osmium tetroxide) for 1 h at 4 °C and rinsed in the same buffer. Dehydration in a graded ethanol series from 60% to 99%, was followed by embedding in Epon-Araldite with propylene oxide as bridging solvent. Thin sections were taken with a diamond knife on a LKB Bromma ultramicrotome (LKB^®^, Bromma, Sweden), and mounted on formvar coated 50 mesh grids. Then, sections on grids were stained with uranyl acetate (20 min, room temperature) and lead citrate (5 min, room temperature). Finally, the sections were investigated with a Philips^®^ EM 208 (Thermo Fischer Scientific, Hillsboro, OR, USA). Digital pictures (1376 × 1032 pixels, 8b, uncompressed greyscale TIFF files) were obtained using a high-resolution digital camera MegaViewIII (SIS^®^, Muenster, Germany) connected to the TEM.

EAG Analysis. Electrophysiological experiments were performed to evaluate the olfactory response of *S. zeamais* antennae to essential oils of *C. nubigeum* and *C. tomentosum*. Octanal (99%) (Sigma Aldrich, St. Louis, MO, USA) was used as reference standard stimulus to normalize the electrophysiological responses. To prevent rapid evaporation of test compounds and for the dose-dependence experiments, odor stimuli were dissolved in paraffin oil (Fluka, Buchs, Switzerland). *S. zeamais* adults were anaesthetized by refrigerating them at 4 °C for about 120 s. A single insect was inserted into the end of a truncated plastic pipette tip allowing the insect head to come out of the tip. The insect was immobilized inside the pipette with the help of Patafix (UHU Bostik, Milan, Italy) and the tip of the rostrum was carefully excised for about half of its length using micro scissors to allow the contact with the reference electrode. The pipette with the insect was placed on a dental wax base close to a coverslip portion and the insect head was fastened to the dental wax base by a tungsten hook. One antenna was fixed in place by double-sided tape to the coverslip portion to access the antennal club tip. Electrical activity was recorded by inserting the reference electrode into the rostrum, while the tip of the recording electrode was connected to the edge of the specialized elliptical area in the distal part of the apical antennomere. Glass capillary electrodes (1.5 mm outer Ø, 0.9 mm inner Ø) filled with Ringer solution [35], containing 5 g/L of polyvinylpyrrolidone (Fluka), in contact with a silver wire were used. The capillary tubes were drawn to a fine point using a microelectrode puller PC-10 (Narishige, Tokyo, Japan) to get an inner diameter wide enough to enable the contact with a small portion of the club. The analog signal was detected through a probe with a high-input impedance preamplifier (10×) (EAG Kombi-probe, Syntech, Germany), captured and processed with a data acquisition controller (IDAC-4, Syntech, Germany) and analyzed using EAG 2000 software (Syntech, Germany).

For a screening of the EAG responses, test compounds diluted as 10% solution *v*/*v* in paraffin oil were delivered as 10 µL samples placed on a filter paper (15 mm × 15 mm). The impregnated filter paper was placed into a glass Pasteur pipette (150 mm length) constituting an odor cartridge. The control stimulus consisted of a similar pipette containing a filter paper impregnated with 10 µL aliquot of paraffin oil. Fresh stimulus pipettes were prepared every day. The tip of the glass pipette was placed about 3 mm into a small hole in the wall of a L-shaped glass tube (130 mm long, 12 mm diameter) oriented towards the antennal preparation (5 mm away from the preparation). The stimuli were provided as 1 s puffs of purified, charcoal-filtered air into a continuous humidified main air stream (up to 60% RH obtained by bubbling air through a washing glass bottle) at 2500 mL/min, that was flowing over the antennal preparation at a speed of 50 cm/s generated by an air stimulus controller (CS-55, Syntech, Germany). At least a 1 min interval was allowed between successive stimulations for antenna recovery. The two essential oils of *C. nubigeum* and *C. tomentosum* were furthermore tested to determine dose-dependent responses at four different concentrations *v*/*v* in paraffin oil (0.1%, 1%, 10%, 20%), using a 10 µL solution starting with the lowest concentration to minimize olfactory adaptation. To evaluate EAG responses, the maximum deflection of the recorded EAG signal after stimulation with an odor was used. For dose-dependent response analysis, *S. zeamais* sensitivity to the different chemical compounds was recorded as a percentage of all recorded EAG responses to octanal at 10% *v*/*v* used as reference standard stimulus. This allows compensation for changes of the sensitivity of an antenna during the course of an experiment and the comparison between experiments performed with antennae of different sensitivities. After the EAG test, each insect was dissected to be sexed. The tests were conducted on 13 females and 17 males. For dose-dependent response, the dose sequence was repeated 2–4 times for each individual, using the mean values of each individual for the analysis. All the experiments were conducted at room temperature (23 ± 2 °C).

### 2.5. Statistical Analyses

The proportion of individuals choosing the EOs-treated chamber, in the behavioral assays were compared by likelihood chi-square test, with a null hypothesis of a 50:50 chance of insects choosing the control versus the EOs-treated chamber (SPSS 22.0, IBM SPSS Statistics, Armonk, North Castle, NY, USA). The median repellent concentration (RC_50_) of the EOs against *S. zeamais* adults was calculated by log-probit regressions. Significant differences between the RC_50_ values of the two EOs were determined by relative median potency (RMP). Differences between the RC_50_ values were considered statistically significant when RMP 95% confidence interval was ≠ 1.0 (SPSS 22.0, IBM SPSS Statistics, Armonk, North Castle, NY, USA). Differences in the latency time and the permanence time were analyzed by generalized linear models (GLM, Poisson loglinear model) by assessing the main effect of EO and concentration as predictors and calculating the estimated marginal means (EMMeans) (SPSS 22.0, IBM SPSS Statistics, Armonk, North Castle, NY, USA). EAG absolute responses (mv) of the two gender and to *C. nubigeum* and *C. tomentosum* essential oils, octanal and paraffin oil as control were compared by two-way analysis of variance (ANOVA) considering the gender and the odor compound as main factors. F tests were used to assess the significance of the effects and their interactions. For significant factors, the Dunnett test was used to compare the response to each compound to those of the control (paraffin oil) [36]. Data obtained in the dose-response experiments and reported as relative responses to octanal (%) were analyzed separately for *C. nubigeum* and *C. tomentosum*, by two-way analysis of variance (ANOVA) considering the gender and the concentrations as main factors. and by the unequal N Tukey honestly significant difference HSD test for multiple comparisons between the means [36]. For significant factors, the Dunnett test was used to compare the response to each concentration to those of the control (paraffin oil) [36]. Before the analysis, Box–Cox transformations were used to reduce data heteroscedasticity [37]. Analysis were performed by the SPSS 22.0 software (IBM SPSS Statistics, Armonk, North Castle, NY, USA).

## 3. Results

### 3.1. Essential Oils Compositions

The complete compositions of the essential oils extracted from flowering aerial parts of *Clinopodium nubigenum* and *C. tomentosum* are reported in Table 1. Oxygenated monoterpenes represent the most abundant chemical class of compounds in both the EOs, accounting for up to 74.0 and 96.2% in *C. nubigenum* and *C. tomentosum*, respectively. The identified compounds belonging to this class, though, are quite different in the two species. Carvacrol is the most abundant oxygenated monoterpene (and overall compound) in *C. nubigenum* EO, whilst it was not detected in the *C. tomentosum*. Conversely, isomenthone accounts for over 45% in *C. tomentosum*, with the highest detected relative abundance in this EO; its presence in *C. nubigenum* is still relevant (6.4%), but it is significantly less represented. Pulegone follows as the second most abundant volatile organic compound (VOC) in both EOs. Piperitenone and its epoxidized derivative were only detected in *C. tomentosum*. Monoterpene hydrocarbons showed a far more relevant relative abundance in the composition *C. nubigenum* EO (19.7% vs. 1.9% in *C. tomentosum*). Among them, *p*-cymene and γ-terpinene showed the highest contents (9.1 and 5.3%, respectively), whilst their presence was not detected in *C. tomentosum* EO. Non-terpene derivatives were also more represented in the composition of *C. nubigenum*, with 3-octanol and 1-octen-3-yl acetates as the main ones.

### 3.2. Behavioural Assays

**Figure 1:**
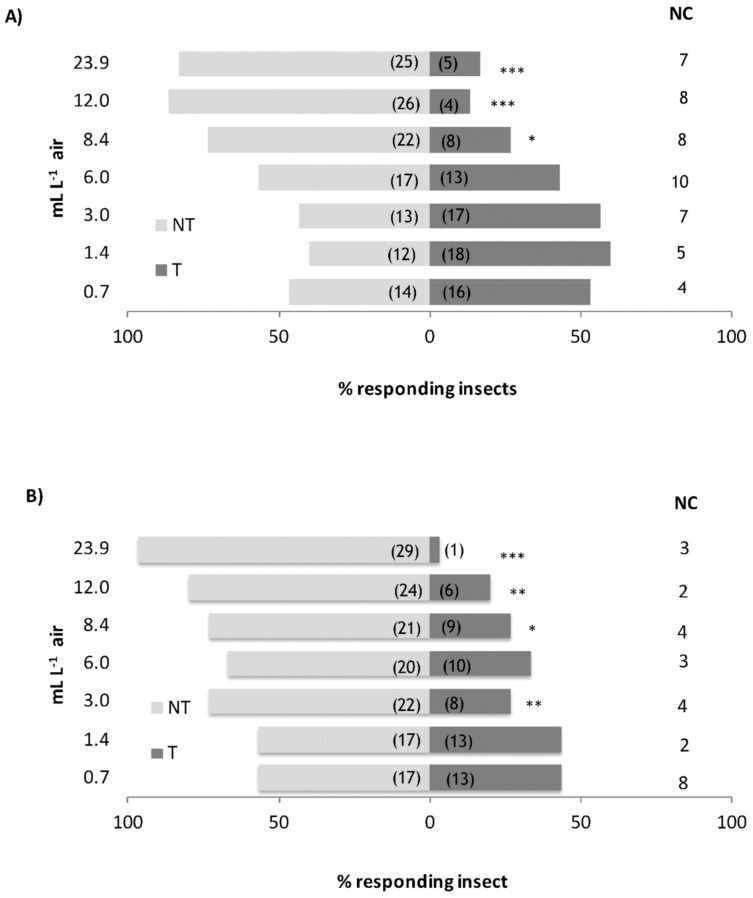
Behavior of adults of *Sitophilus zeamais* in the presence of *Clinopodium nubigenum* and *C. tomentosum* essential oils (EOs). Histograms represent the percentage of insects that chose the cue or the control chamber. In brackets, the number of insects that made the choice. NT, % of insects that chose the control chamber; T, % of insects that chose the EO treated chamber; NC, number of non-choosing insects. Each EO was tested vs. clean filter paper in two-choice bioassays. Asterisks indicate significant differences in the number of the choosing insects (*χ*^2^ test; *, *p* < 0.05; **, *p* < 0.01; ***, *p* < 0.001). (**A**), *C. nubigenum* EO; (**B**), *C. tomentosum* EO.

*S. zeamais* adults showed negative chemotaxis to the EOs. We observed a significant repellent effect of the EOs starting from the concentration of 8.4 mL L^−1^ air both for the EO of *C. nubigenum* (*χ*^2^ = 6.533, df = 1, *p* = 0.011) and for the *C. tomentosum* one (*χ*^2^ = 4.800, df = 1, *p* = 0.028) (Figure 1A,B). The RC_50_ calculated by probit regression indicated a stronger repellent effect of *C. tomentosum* EO than the *C. nubigenum* one. Actually, the repellent activity by the EO of *C. tomentosum* (RC_50_ = 6.579 μL L^−1^ air) was about twice stronger than the *C. nubigenum* one (RC_50_ = 11.216 μL L^−1^ air) (Table 2) and, according to the RMP analysis, such difference can be considered as statistically significant [*C. tomentosum* vs. *C. nubigenum* RMP = 0.497 (0.267–0.813)]. The latency time and the time of permanence on the stimulus (Table 3) were analyzed using the generalized linear model (GLM). The GLM regressions were overall significant (omnibus test, *p* < 0.001). A significant difference was found for the latency time between the EOs (Wald *χ*^2^ = 10.137, *p* = 0.001) with a more ready response to the *C. tomentosum* than the *C. nubigenum* EO (EMMeans = 117.290 ± 0.747 and 113.951 ± 0.737, for *C. nubigenum* and *C. tomentosum*, respectively). On the contrary, no difference in the permanence time of *S. zeamais* was observed between the two EOs (Wald *χ*^2^ = 0.455, *p* = 0.500) (EMMeans = 130.481 ± 0.788 and 129.731 ± 0.786, for *C. nubigenum* and *C. tomentosum*, respectively).

### 3.3. Antennal Morphology

**Figure 2:**
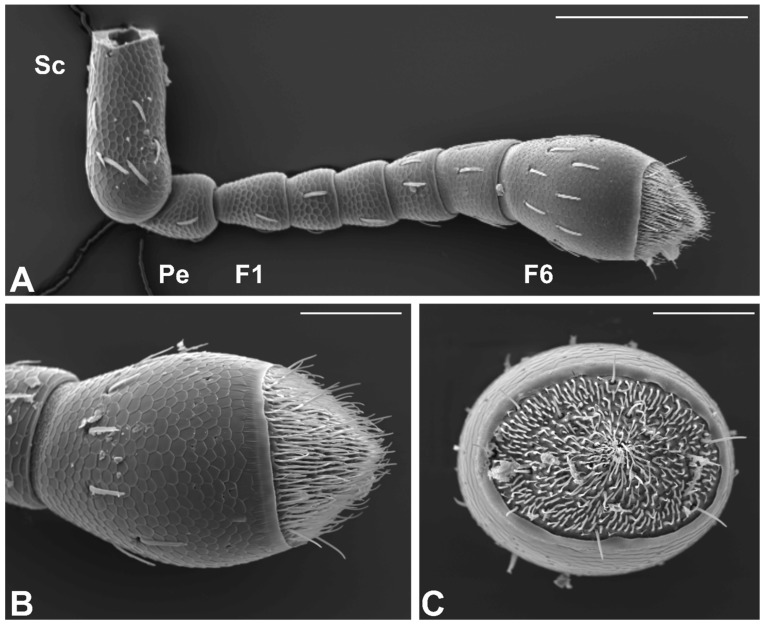
Scanning electron microscopy (SEM) micrographs of a female Sitophilus zeamais antenna. (**A**) General view of the whole antenna. The apical club-like antennomere is clearly visible on the right side of the picture. (**B**,**C**) detail of the apical antennomere in the lateral (**B**) and apical (**C**) view, respectively. The apical sensory area is clearly visible. F1, first antennomere of the funicle; F6, sixth antennomere of the funicle; Pe, pedicel; Sc, scape. Bar scale: (**A**) 200 µm; (**B**,**C**) 50 µm.

General Description. In *S. zeamais*, the antennae are of the geniculate type, typically inserted laterally on the basal part of the head capsule. During normal activity, insect antennae are brought in a forward or lateral position. In both male and female, the antenna is about 850 µm long, and is composed of a total number of eight antennomeres (Figure 2A). The first one is the scape (connecting the antenna with the head capsule), which is the most developed antennal segment in terms of length (about 250 µm long). The scape is followed by the pedicel (about 75 µm long) and the flagellum, which is composed of six antennomeres. The proximal five flagellomeres are sub-cylindrical in shape, and together measure about 300 µm in length. However, the most elongated one is actually the last antennal segment, measuring about 200 µm in length. It is characterized by a cylindrical shape, with a conical, pointed apical part (Figure 2A,B).

The cuticular surface of the whole antenna appears as finely sculptured, with the presence of a single type of structures; these appear as flattened setae, distributed in variable number along the antennomeres, including the apical one. The most interesting part of the antennae is, however, the distal part of the apical antennomere, which is defined by a specialized area, elliptical in shape (if observed from the top) (Figure 2C). This area presents quite a number of sensory structures of various type and shape. It is noteworthy that no differences occurred as regards the general structure of the antenna and the presence and distribution of the sensory structures. Ultrastructural investigations revealed the presence of five different types of antennal sensory structures. In order to map the sensilla, at least six SEM photographs were taken at a given magnification (3000×) in order to picture the whole apical part of the antenna. These images were composed together into a single, high magnification picture (12,000 × 5000 dpi, 8 bit greyscale) using the software Photoshop^®^ CC 2018 (Adobe Systems Incorporated, San Jose, CA, USA). Pictures were analyzed using the software ImageJ (NHI, Open Source). The data about the number and size of the different mapped sensory structures, described in detail in the section below, is reported in Table 4.

**Figure 3:**
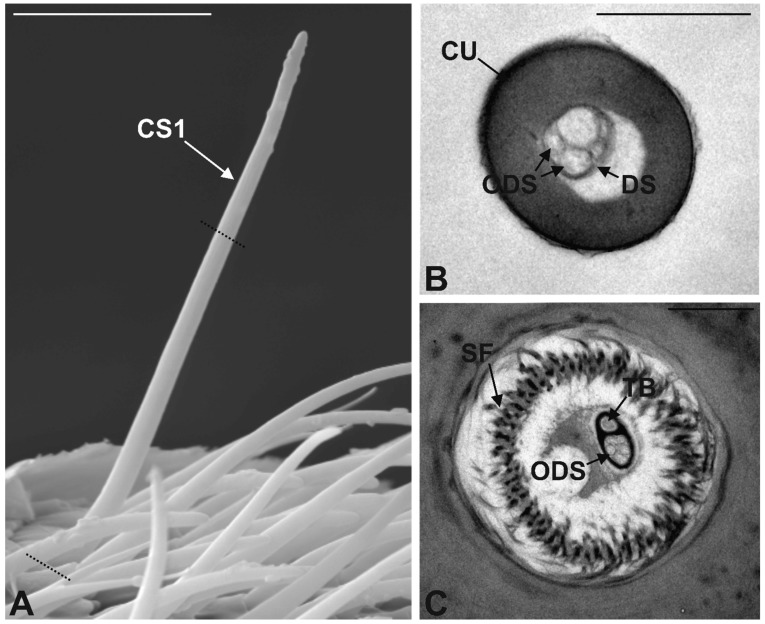
Chaetic Sensilla Type 1 (CS1). (**A**) SEM micrograph showing an CS1 sticking out from the rest of the antennal sensilla. Dotted lines represent the section planes showed in Figure 3B,C, respectively. (**B**) Transmission electron microscopy (TEM) cross section of CS1, the cuticle (CU) appears thick, the internal lumen is partly occupied by five outer dendritic segments (ODS) enveloped by a dendrite sheath (DS). (**C**) TEM section taken at the socket level, showing the large socket rich in suspension fibers (SF) and the outer dendritic segments (ODS) grouped by the dendrite sheath into two separated spaces, one occupied by five neurons and the other by a single neuron ending in a tubular body (TB). Bar scale: (**A**) 10 µm; (**B**) 0.1 µm; (**C**) 1 µm.

Chaetic Sensilla Type 1 (CS1). These sensilla are characterized by a straight cuticular shaft inserted on to the antennal wall with a variable angle, but normally exceeding 45° (Figure 3A). CS1 are characterized by the absence of wall pores, the longitudinally grooved cuticle and by the presence of a wide basal socket, through which the sensillum in inserted on the antennal wall. They were found to occur in various parts of the sensory area, i.e., at the level of the inferior margin as well as at the tip. CS1 represent the less abundant sensilla occurring on the sensory area (see Table 4). Serial TEM cross sections of CS1 cuticular shaft revealed the presence of five unbranched sensory neurons enclosed in a single dendritic sheath (Figure 3B). This bundle of dendrites runs inside the sensillum up to the tip. At its base, CS1 is equipped with an elaborated flexible socket, which suspends the sensillum around the antennal wall. A mesh of suspension fibers was observed, as well as the presence of a sixth sensory neuron, which ends at the socket level in a typical tubular body (Figure 3C).

**Figure 4:**
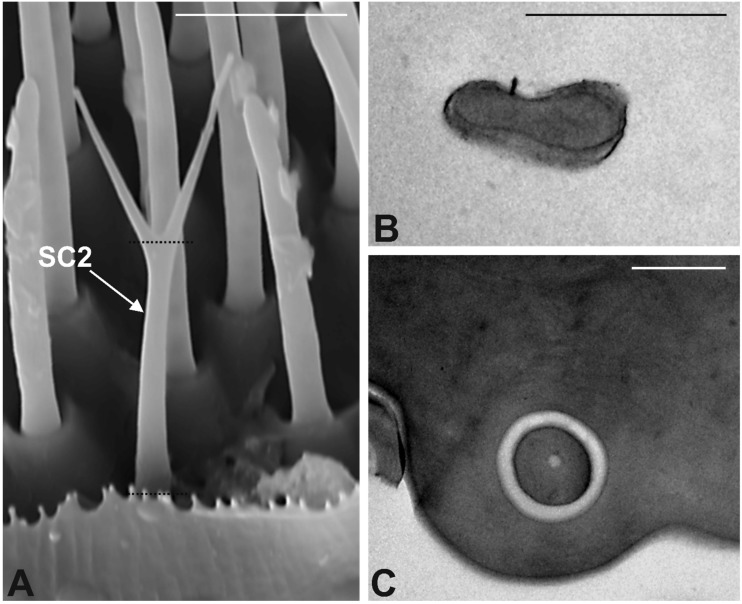
Chaetic Sensilla Type 2 (CS2). (**A**) SEM dorsal view of a CS2 showing the distal part divided into two pointed branches. (**B**,**C**) TEM micrographs showing cross section of an CS2 taken at the level of the dotted lines reported in A. In both cases the sensillum shows a thick and compact cuticle and the absence of sensory neurons entering the sensillum. Bar scale: (**A**) 5 µm; (**B**,**C**) 1 µm.

Chaetic Sensilla Type 2 (CS2). The sensilla belonging to this class are typically characterized by a branched structure, i.e., the distal part of the cuticular shaft is divided into 2–4 branches ending with a sharp tip (Figure 4A). These sensilla are inserted on the antennal wall through a socket according to an angle below 30°, therefore CS2 do not protrude above the antenna profile. The cuticular wall appears smooth and poreless. We counted about 40 CS2 per antenna, and, similarly to what we observed for CS1, they are comparatively less numerous than the other sensilla type we described. CS2 are distributed randomly on the whole apical sensory area, although they are preferentially found along the lower margin. TEM sections revealed the presence of a thick cuticle without sensory neurons (Figure 4B,C). At their base, CS2 revealed the presence of a single sensory neuron ending in a tubular body.

**Figure 5:**
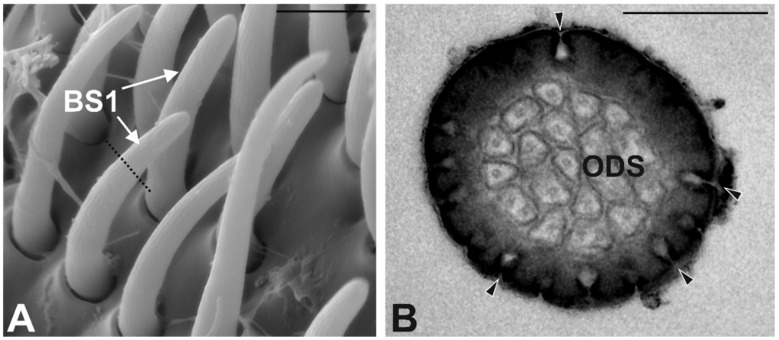
Basiconic Sensilla Type1 (BS1). (**A**) SEM micrograph showing a bunch of SB1 with the typical curved shape. Thin cuticular grooves longitudinally oriented can be observed. The dotted line represents the section plane reported in (**B**). (**B**) TEM cross section of BS1, with evidence of numerous cuticular pores (arrowheads) distributed all over the surface and corresponding to the external cuticular grooves. The internal lumen is completely occupied by branched outer dendritic segments (ODS). Bar scale: (**A**) 5 µm; (**B**) 0.5 µm.

Basiconic Sensilla Type 1 (BS1). They are typical “peg” sensilla, with an average length of about 12 μm. The cuticular shaft is slightly curved and finely grooved for all of its length, except for the basal part, where the cuticle appears smooth (Figure 5A). BS1 end with a rounded tip and are inserted on the antennal wall through a rigid socket. This type of sensilla was often found grouped in some regions of the apical sensory area, forming clusters of sensilla. Because of their length and their curved shape, BS1 are arranged almost parallel to the antennal profile, often hidden by other type of sensilla. BS1 are the most abundant sensilla type. TEM investigations revealed the presence of a cuticular wall, with pores (diameter about 30 nm) distributed all over the surface. The sensilla lumen is completely filled with the dendritic branches of the associated sensory neurons (Figure 5B).

**Figure 6:**
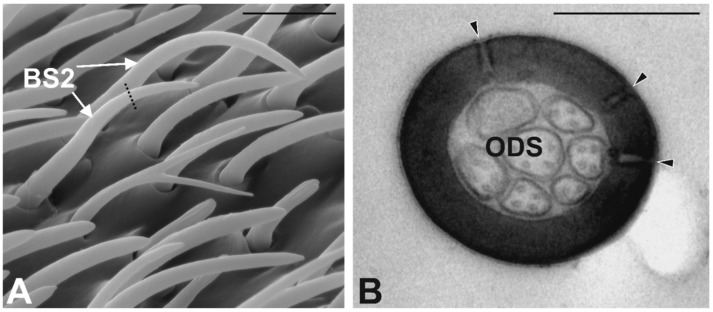
Basiconic Sensilla Type2 (BS2). (**A**) SEM micrograph showing BS2 sensilla, appearing longer than the surrounding sensilla and distinctly curved. Dotted line corresponds to the TEM section plane. (**B**) TEM micrograph showing a cross section of BS2. The cuticle is thick and perforated by few pores (arrowheads). The sensillum lumen is filled by branched outer dendritic segments (ODS). Bar scale: (**A**) 5 µm; (**B**) 0.5 µm.

Basiconic Sensilla Type 2 (BS2). These sensilla show an elongated and strongly curved at the distal half level cuticular shaft (Figure 6A). They are inserted on the antennal wall through a socket analogous to SB1, with a starting angle of about 35°. The distal half of the sensilla is curved downward, therefore following the curvature of the antennal wall. Because of these features, SB2 are positioned above the other type of sensilla, except for the SC1. The antennal cuticular wall is smooth, and the apical part is pointed. SB2 are distributed quite evenly at the level of the sensory area, with a preference for the apical part of the antenna; they represent the second most abundant type of sensilla. Ultrastructural details reveal the thick cuticle, where a few pores open (Figure 6B). The cuticle is thicker and less porous, quite different from the cuticle we described for SB1. TEM sections taken at the medial sensillum region revealed the presence of branched dendritic processes that fill the sensillum lumen.

**Figure 7:**
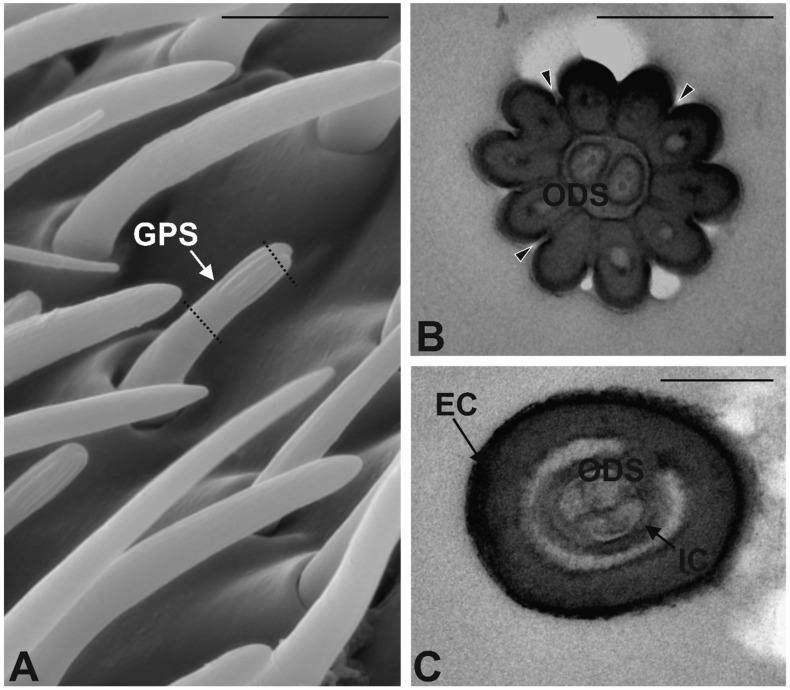
Grooved Peg Sensilla (GPS). (**A**) SEM micrograph showing an GPS sensillum characterized by the presence of finger-like projections in the apical part. Dotted lines correspond to the TEM section planes. (**B**,**C**) TEM cross section taken at the apical and proximal region, respectively. In (**B**) the sensillum shows a cuticle organized in thick cuticular elements corresponding to the external finger-like projections. At the level of the grooves several pores can be observed crossing the whole cuticle. The internal lumen is occupied by two outer dendric segments (ODS). In C the section is taken at a lower level and shows a typical double-walled organization, with the external cuticular wall (EC) and the internal cuticular wall (IC) enclosing three outer dendritic segments. Bar scale: (**A**) 5 µm; (**B**,**C**) 0.5 µm.

Grooved Peg Sensilla (GPS). GPS are small sensilla evenly distributed all over the antennal sensory area. They are inserted on the antennal wall through a rigid socket: Their base is inserted almost orthogonally, whilst the whole structure is bent downward and runs almost parallel to the antennal wall. Distally, the cuticular shaft appears clearly grooved, giving rise to digitiform cuticular elements reaching the rounded tip (Figure 7A). The basal part of the cuticular shaft is smooth. GPS sensilla are often found interspersed within BS1 and BS2. TEM cross sections revealed the actual structure of the sensillum, with the typical double-walled organization. Apically, 10–12 cuticular ridges are present, while the proximal half of the cuticular shaft is smooth externally. Spoke channels are found between the ridges (Figure 7B). Cross and longitudinal sections show, distally, 2 sensory neurons entering the lumen without branching. Proximally, each sensillum presents 2 cuticular chambers: The innermost chamber is occupied by the outer dendritic segments of three sensory neurons (Figure 7C).

**Figure 8:**
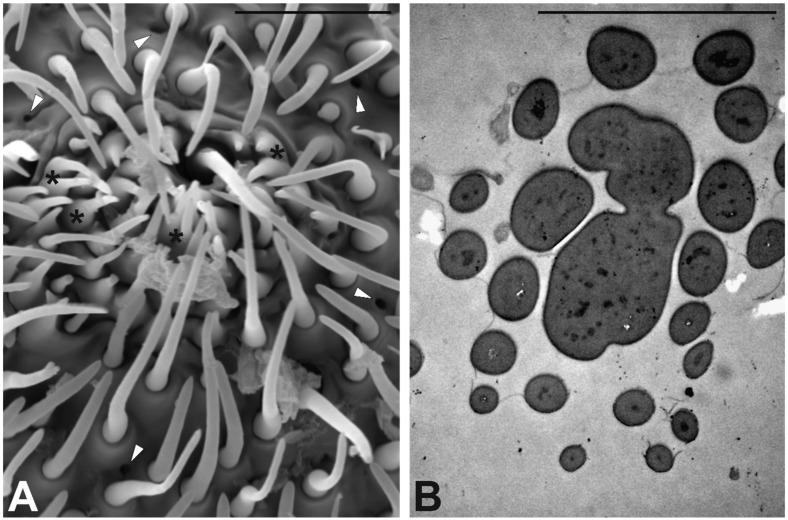
(**A**) SEM micrograph showing the apical part of the last antennomere (F6) in S. zeamais female. Several cuticular structures (*) are grouped around the tip, together with numerous sensilla. Several cuticular pores (white arrowheads) are present. (**B**) TEM micrograph showing a cross section of the antenna tip: The structures are made of solid cuticle without sensory neurons associated. Bar scale: 5 µm.

Besides the aforementioned sensilla, the analysis of the antennal surface revealed peculiar structures concentrated at the very tip area of the antenna. These structures show a wide, ampulla-like base rising from the antennal wall without an apparent socket. The basal part continues as an elongated, thin and pointed structure (Figure 8A). TEM cross sections taken at this level revealed the presence of solid cuticle without sensory neurons associated (Figure 8B). We also highlighted the presence of series of cuticular pores (about 100), evenly distributed (Figure 8A). Ultrastructural observations revealed the absence of “peg in pit” or “coeloconic” sensilla (typically hidden below the cuticle and communicating with the surface through a small opening), while the pores could be associated with glandular structures.

### 3.4. EAG Analysis

**Figure 9:**
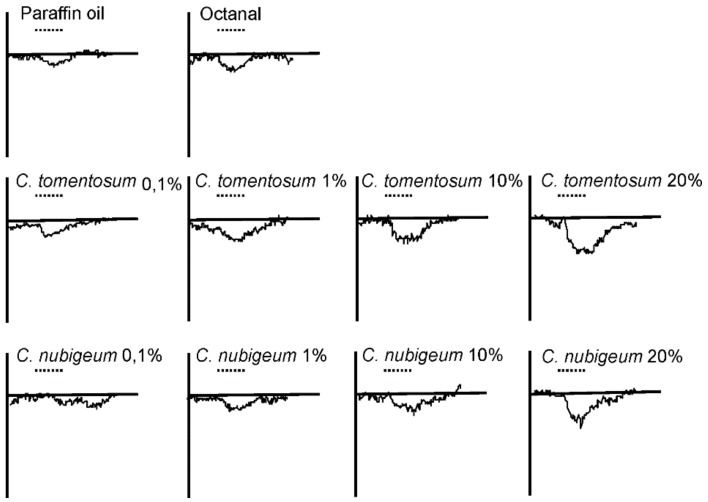
Examples of electroantennography (EAG) response waveforms of *Sitophilus zeamais* to 10 µL of the different compounds at different concentrations, compared with the response to the controls (paraffin oil). Dotted line corresponds to 1 s stimulus.

**Figure 10:**
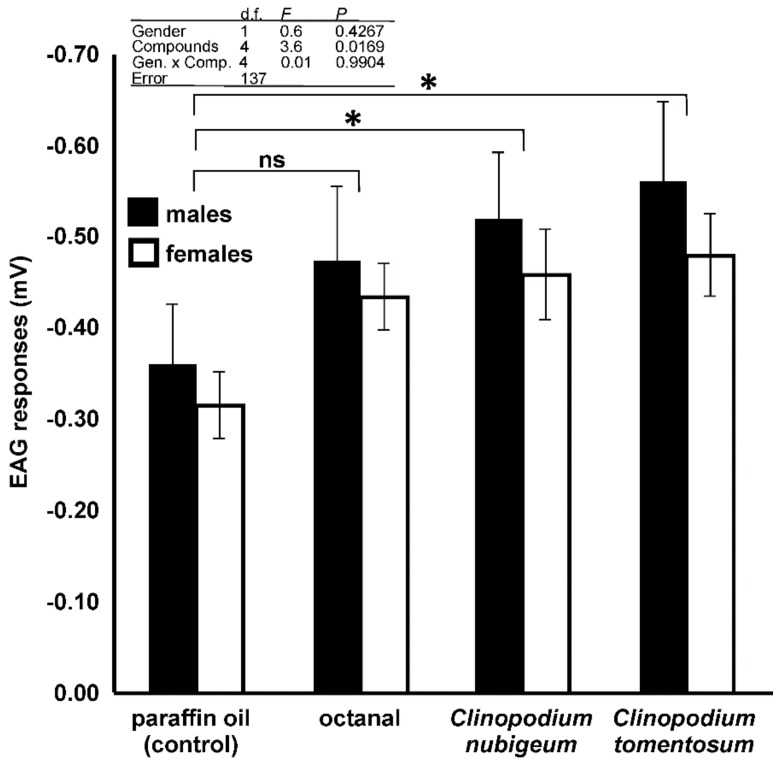
EAG responses (mV) of male and female antennae of *Sitophilus zeamais* to compounds dissolved in paraffin oil at 10% (v/v) concentration. Bars indicate the mean ± standard error. Table inset shows the statistical parameters of two-way ANOVA. The response of the two gender to each compound is compared to those of the control (paraffin oil) (*, *p* < 0.05; ns, not significant; ANOVA, Dunnett post hoc test).

**Figure 11:**
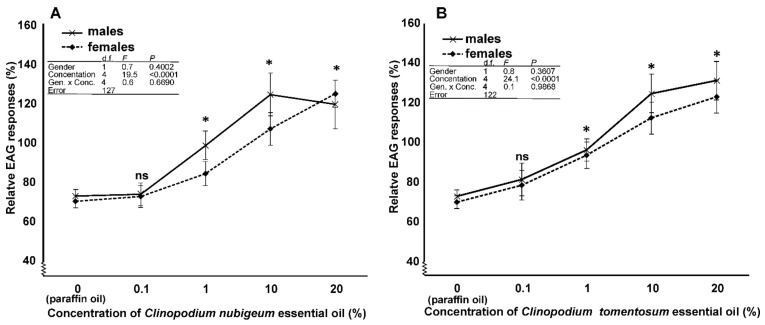
Dose–response relationships for stimulation of male and female antennae of Sitophilus zeamais with Clinopodium nubigenum (**A**) and C. tomentosum (**B**) essential oils. Data (mean ± standard error) represent the EAG response relative to octanal (%). Table insets show the statistical parameters of two-way ANOVA. The response of the two gender to each compound is compared to those of the control (paraffin oil) (*, *p* < 0.05; ns, not significant; ANOVA, Dunnett post hoc test).

Depolarizing EAG responses were recorded in both males and females (Figure 9). The EAG responses to 10% concentration of the different compounds tested were not statistically different between the two genders, but they were different among the compounds. The interaction between the genders and the various compounds was not statistically significant (Figure 10 table inset). In particular, the responses to *C. nubigeum* and *C. tomentosum* EOs were higher than those to paraffin oil (Figure 10). In the experiments to determine dose-dependent responses to both *C. nubigeum* and *C. tomentosum* EOs, the EAG responses were not statistically different between the insect genders, but they were different among the concentrations. The interaction between the sexes and the concentration was not statistically significant (Figure 11A,B table inset). In particular, for both the EOs, the response to 0.1% concentration was not statistically different from the control, while the responses to 1, 10 and 20% concentrations were higher than the control (Figure 11A,B).

## 4. Discussion

To date, the control of foodstuff pests has been mainly based on synthetic insecticides and fumigants, however, since their use has raised food and environmental safety concerns, alternative treatments are highly demanded. EOs are regarded as very promising substances for the formulation of low-toxic, eco-friendly pest control products. In this context, the huge richness of the Andean flora may represent an important source of bioactive substances. In this study, we report the behavioral effects of *C. nubigenum* and *C. tomentosum* EOs on the maize weevil *S. zemais*. While *C. nubigenum* EOs were already tested for their toxic effect as well as oviposition deterrent effect on *L. sericata* [17], this is the first report showing a repellent effect of *C. tomentosum* against insects. We also made a chemical characterization of both *Clinopodium* species to highlight differences. We report a complete mapping of the antennal structures in *S. zeamais* with a characterization of the olfactory sensilla, and we also tested the antennal activity of different concentration of *C. nubigenum* and *C. tomentosum* EOs.

### 4.1. Chemical Characterization of EOs

As reported in the literature, monoterpenes are the most relevant compounds detected in the composition of *C. nubigenum* EO, mainly in their oxygenated form. All the described *C. nubigenum* EOs were extracted from plants gathered on the Andean highlands of Ecuador, and quantitative differences emerged from these samples, probably depending on the latitude of collection. The specimens studied in the present work were gathered on the mountains of the Imbabura region, which is situated in northern Ecuador. Noriega et al. studied the composition of the EO extracted from plants collected in the Pichincha province, situated in a slightly more central area of Ecuador. Carvacrol and its acetate were the most abundant compounds in the composition, with the latter, which was not detected in the sample of the present study, accounting for over 40% of the total composition (vs. 21.21% of the former) [38]. Pulegone was far less represented (6.09%) compared to our sample, whilst thymol, which accounted for over 5.5% in Noriega et al., was not detected in our EO. Ruiz et al. collected their plant samples in Piedra Bola, in the Loja region, situated in the central area of Ecuador. Similarly to Noriega et al., carvacrol and its acetate were the most abundant compounds evidenced in the EO. Moreover, thymol showed a significant relative abundance, whilst pulegone relative content went down to 1.9% [39]. The farthest EO, both in terms of geographical provenience of the plant material and composition, is the one reported in Gilardoni et al. [25]. Their *C. tomentosum* specimens were collected in the Saraguro territory of the Loja province, in southern Ecuador. Their EO was dominated by pulegone, which accounted for over 70% of the total composition. Carvacrol was not detected, whilst linalool was found with a relative abundance higher than 7% [25], whilst it accounted only for 0.1% in our sample. To the best of our knowledge, only one study reported *C. tomentosum* EO composition. Consistently with our findings, Benzo et al. detected isomenthone and pulegone as the most abundant compounds in the EO extracted from *C. tomentosum* collected near river Chambo, in central Ecuador; moreover, piperitenone was over 1.8% [40]. Conversely, menthone was significantly more represented in their EO (6.6%) compared to our sample.

### 4.2. Behavioural Assays

In this study we observed an increasing repellent effect of the EO of *C. nubigenum* and *C. tomentosum* EOs against *S. zeamais* adults, in relation to increasing concentration of the EOs. This is in agreement with the results of the EAG analysis. This suggests an insect olfactory response to at least some of the EOs compounds, which releases a repellence behavior. In line with these results, a clear repellence against *S. zeamais* adults was also observed by Bougherra et al. [11] for the EO extracted from *Pistacia lentiscus* L. (RD_50_ values ranging from 0.010 L cm^−2^) who reported that *S. zeamais* was overall more susceptible to the *P. lentiscus* EO than *Rhyzopertha dominica* (Coleoptera: Bostrichidae), *S. zeamais*, and *Tribolium confusum* (Coleoptera: Tenebrionidae). In line with our results, *S. zeamais* exhibited a negative chemotaxis also in the presence of the EO extracted from two chemotypes of *Foeniculum vulgare* Mill., with RD_50_ of 0.176 and 0.124 mg cm^−2^, respectively. In our trial we observed a higher repellence of the EO of *C. tomentosum* than the EO of *C. nubigenum* even if *S. zeamais* showed a more ready reaction (shorter latency time) to the EO of *C. nubigenum* compared to that of *C. tomentosum*. Such differences in the efficacy and latency time may be due to the different chemical composition of the EOs whose bioactivity depend not only but also on to the combined action of their single chemical constituents [41] with synergistic and antagonistic effects on the insect behavior [12].

### 4.3. Antennal Sensilla in S. zeamais

The antennae of *S. zeamais* are made of a distal club that presents apically a specialized sensory area. Indeed, most of the antenna sensilla are located at the level of this area, while the rest of the antennal sensilla are sparsely distributed on the other antennomeres. This is particularly true if we restrict our observation to the olfactory sensilla. Among weevils, a similar organization of the antenna was reported in *Rhynchophorous palmarum* L., where the sensory area was restricted to the apical, remarkably modified apical antennomere [42]. The antennae of Sitophilus spp. were studied and analyzed in previous papers, however most of the reported data were obtained only using scanning electron microscope. Abd El-Ghany and Abd El-Aziz (2017) [28] reported the occurrence of antennal sensilla in *S. granarius*, but only few SEM pictures are shown, while in a previous paper in the same species, Ali et al. [26] analyzed the antennal sensilla using bot SEM and TEM approach. However, the information reported in this paper are rather confused and supported by incomplete TEM data. To date, this is the first contribution to the study of antennal sensilla in Sitophilus spp. with a comprehensive analysis of the different antennal sensilla. In *S. zeamais* there is no sexual dimorphism as regards the general structure of the antennae (shape and size of the antennomeres, number of antennal sensilla) and the type and distribution of the different antennal sensilla. Antennal sexual dimorphism was reported for species belonging to different insect orders, with different biological significance. In Hymenoptera, antennal dimorphism can occur either in terms of antennal shape (i.e., filiform in males vs. clubbed in females) or presence of specialized antennomeres housing pheromone glands (mostly common in males) ([43] and refences therein). In Lepidoptera relying on long-distance communication mediated by female-produced sex pheromone, males often possess modified bi-pectinate antennae housing large number of specialized olfactory sensilla [44]. The absence of sexual dimorphism in *S. zeamais* could be related with the relatively narrow involvement of the antennae in the sexual behavior, possibly due to the absence sex-pheromone based communication. Among the five different type of antennal sensilla, Chaetic Sensilla Type 1 (CS1) are numerously less represented than the others but are the most evident because of their length and their wide insertion angle with the antennal wall, that makes them easily visible. This feature is consistent with a contact-chemoreceptive gustatory role for CS1, strongly supported also by the presence of a combination of five chemosensory neuron and a single mechanoreceptive neuron ending with a tubular body. This neuronal scheme is quite common among different insect orders such as: Hemiptera [45,46,47], Diptera [48,49,50], and Lepidoptera [51,52,53]. In regards to Coleoptera, similar sensilla were reported in several species [54,55,56,57], for which a role in the detection of salt, sugar and Ph variation was demonstrated [58,59]. Chaetic Sensilla Type 2 (CS2) are also present in low numbers and are typically characterized by a bi- or multi-partite distal region. The absence of cuticular pores, sensory neurons within the cuticular shaft, presence of a single sensory neuron with tubular body and positioning along the external border of the antenna deals with a possible mechanoreceptive function [60]. Similar sensilla were reported to occur on other weevil species, i.e., *Rhynchophorous palmarum* L. [42] and *Hylastinus obscurus* Marsham [61], for which the same functional hypothesis was made. However, in *Pissodes nitidus* Roel. for the same type of sensilla a functional role as olfactory sensilla was proposed [62], based on misleading interpretation of the presented TEM data.

The main olfactory organs in *S. zeamais* are Basiconic Sensilla Type 1 (BS1) and Type 2 (BS2), which makes also the two most represented classes of antennal sensilla in this species. The proposed olfactory function is strongly supported by ultrastructural evidences, i.e., the presence of porous cuticular wall and dendritic branches. Given this, their possible involvement in the perception of volatile chemicals, including EOs extracts, is highly probable. In *R. palmarum*, Type IV and V sensilla were reported as olfactory sensilla on the basis of their external and internal morphological features [42], strongly resembling Basiconic Sensilla 1 and 2. We found that antennal sensilla distribution does not follow a particular pattern, with most of the sensilla distributed evenly on the sensory area (with the exception of CS2 located along the outer edge). This organization of olfactory sensilla could be related with was found in other weevils, where the absence of distinct area bearing specific sensilla types is quite a normal feature. As already suggested by Said et al. [42], the hypothesis of a mosaic-like organization of olfactory sensilla in regards to their functional response to different volatiles could apply to *S. zeamais* as well, with different olfactory receptor neurons from the same sensilla type responding to different volatiles.

Grooved peg sensilla (GPS) are among the less represented sensilla on the antennae of *S. zeamais*. While their presence was completely neglected in the previous studies, here we report for the first time their presence. Morphologically speaking, GPS show the same structural features already reported for other insect species, for which a role in the detection of volatiles [63,64,65] as well as a function such as thermo/chemosensory receptors or thermo-hygroreceptors [66].

Besides sensory structures, we reported also the presence of cuticular pores located at the level of the specialized sensory area, mostly located very close to the socket of the sensilla but without a specificity for the different sensilla type. Our investigation did not allow us to characterize such structures, although they are very similar to openings of epidermal glands belonging to the third class, according to Quennedey (1998) [67]. Antennal glands were reported to occur on different species and with very different functions, ranging from courtship behavior [43,68] to appeasement of ants in myrmecophilous beetles [69]. In *S. zeamais* we found cuticular pores in both sexes, therefore, a possible role such as pheromone-producing glands should not be considered. In this species we hypothesize a role for antennal glands in maintaining fully operational the sensilla, releasing a protective and/or lubricant secretion helping with removing and preventing dust particles accumulating on the sensilla, considering the dusty environment (grain storage facilities) visited by this species. However further investigations are needed to unveil the role of these glands in *S. zeamais*.

### 4.4. EAG Analysis

Stimulation of the antenna with different concentration of EOs from *C. nubigenum* and *C. tomentosum* elicited a response in both sexes of *S. zeamais*. EAG tests revealed a difference in the antennal response when exposed to 10% concentration of the EOs from both *Clinopodium* species. In the concentration-dependent response test, *S. zeamais* antennal activity showed an increasing response at the increase in the concentration of the EOs, starting with 0.1% of concentration. Despite the differences found in the different components of *C. nubigenum* and *C. tomentosum* EOs, we found a similar EAG response following the increase in concentration of the EOs, and this response was similar in male and females of the weevil. All these data revealed that *S. zeamais* detects the EOs through olfactory sensilla located on the antennae, and that antennal olfactory sensilla are able to detect different concentration of EOs. In a previous study carried out on *S. granarius* and *S. oryzae*, it was demonstrated the ability to discriminate different concentration of propionic acid through antennal olfactory sensilla [70], while our data highlighted for the first time in a member of the Sitophilus genus the antennal sensitivity to EOs.

## 5. Conclusions

The strong demand for eco-friendly and non-toxic products to manage store weevil infestation has been increasing in recent years, leading to a growing interest for the toxic and/or repellent effect of natural products, including plant EOs. Exploiting the potential effect of EOs extracted from indigenous plants could lead to implementation of such products in the control strategies of grain weevil in these areas, avoiding or limiting the use of less sustainable control methods. *C. nubigenum* and *C. tomentosum* EOs showed a strong repellent effect towards adult *S. zemais*, with a clear involvement of the insect antennal olfactory system. However, more studies are needed in order to define the optimal concentration and the best application methods of such substances.

## Figures and Tables

**Table 1:** Complete compositions of the essential oils extracted from flowering aerial parts of *Clinopodium nubigenum* (Kunth) Kuntze and *C. tomentosum* (Kunth) Govaerts.

Constituents	l.r.i. ^a^	Relative Abundance (%)	
		*Clinopodium nubigenum*	*Clinopodium tomentosum*
α-thujene	931	0.9	- ^b^
α-pinene	941	0.5	0.2
camphene	954	tr ^c^	-
sabinene	976	0.4	0.2
1-octen-3-ol	980	tr	-
β-pinene	982	0.5	0.4
myrcene	993	0.3	0.3
3-octanol	993	0.8	-
α-phellandrene	1005	0.2	-
δ-3-carene	1011	tr	-
α-terpinene	1018	1.0	-
*p*-cymene	1027	9.1	-
limonene	1032	1.5	0.9
1,8-cineole	1034	tr	1.1
(*E*)-β-ocimene	1052	tr	-
γ-terpinene	1062	5.3	-
*cis*-sabinene hydrate	1070	0.6	-
terpinolene	1088	tr	-
*trans*-sabinene hydrate	1095	0.1	-
linalool	1101	0.1	0.4
nonanal	1102	0.1	-
1-octen-3-yl acetate	1111	0.9	0.2
β-thujone	1118	tr	-
3-octanol acetate	1124	1.0	-
*cis*-verbenol	1142	-	0.1
isopulegol	1146	tr	-
menthone	1154	-	1.4
citronellal	1155	1.8	-
isomenthone	1164	6.4	48.4
isopulegone	1177	3.5	0.8
4-terpineol	1178	tr	-
α-terpineol	1189	0.2	0.2
myrtenal	1194	-	0.2
decanal	1204	tr	-
citronellol	1230	1.3	-
pulegone	1237	25.4	34.3
neral	1240	-	0.1
piperitone	1252	0.9	4.0
methyl citronellate	1261	-	0.8
geranial	1271	tr	1.1
isobornyl acetate	1285	tr	0.1
*trans*-sabinyl acetate	1291	-	0.1
carvacrol	1298	32.9	-
citronellyl acetate	1350	0.6	-
piperitenone	1351	-	2.1
eugenol	1358	0.8	-
piperitone oxide	1363	0.1	-
piperitenone oxide	1363	-	1.1
α-copaene	1376	0.3	-
β-cubebene	1390	tr	-
β-caryophyllene	1420	-	0.5
γ-muurolene	1477	-	0.5
germacrene D	1478	0.2	-
bicyclogermacrene	1495	0.9	-
(*E*,*E*)-α-farnesene	1507	tr	-
δ-cadinene	1524	0.5	-
spathulenol	1576	0.5	-
manoyl oxide	1988	-	0.8
Monoterpene hydrocarbons		19.7	1.9
Oxygenated monoterpenes		74.0	96.2
Sesquiterpene hydrocarbons		1.8	1.0
Oxygenated sesquiterpenes		0.5	-
Oxygenated diterpenes		-	0.8
Phenylpropanoids		0.8	-
Other non-terpene derivatives		2.8	0.2
Total identified (%)		99.6	100.0

^a^ Linear retention index on a DB5 column; ^b^ Not detected; ^c^ Traces, <0.1%.

**Table 2:** Median repellent concentration (RC_50_) of *Clinopodium nubigenum* and *C. tomentosum* essential oils (EOs) against *Sitophilus zeamais* adults.

EO	RC_50_^a^	Slope	Intercept	*χ*^2^ (df)	*p*
***C. nubigenum***	11.216 (8.762−15.168) ^b^	3.109 ± 0.643	−3264 ± 0.658	4.51 (5)	0.478
***C. tomentosum***	6.579 (4.349−10.833)	1.497 ± 0.306	−1.225 ± 0.259	3.64 (5)	0.603

^a^ Concentration of the extract that repels 50% of the exposed insect; data are expressed as μL L^−1^ air; ^b^ confidence Interval; (df), degrees of freedom; *p*, Pearson goodness-of-fit test.

**Table 3:** *Sitophilus zeamais* latency time and permanence time on the olfactory stimulus (*Clinopodium nubigenum* and *C. tomentosum* EOs) in the two-choice bioassays.

Concentration ^a^	Latency Time		Permanence Time	
	*C. nubigenum*	*C. tomentosum*	*C. nubigenum*	*C. tomentosum*
0.7	120.00 ± 13.36 ^b^	107.23 ± 16.08	131.10 ± 11.29	107.23 ± 14.77
1.4	118.17 ± 14.61	100.23 ± 15.17	116.50 ± 11.42	136.70 ± 18.84
3.0	125.87 ± 13.44	100.27 ± 11.2	123.07 ± 9.25	116.33 ± 14.37
6.0	111.80 ± 13.39	120.70 ± 11.72	123.07 ± 14.34	121.30 ± 15.45
8.4	111.80 ± 13.39	132.70 ± 14.70	123.63 ± 14.34	148.57 ± 14.45
12.0	138.80 ± 15.81	110.80 ± 15.71	143.80 ± 14.66	130.83 ± 15.52
23.9	95.47 ± 12.33	126.57 ± 15.06	154.93 ± 14.45	150.43 ± 19.24

^a^ μL L^−1^ air; ^b^ data are expressed as s (means ± SE); n = 30.

**Table 4:** Morphological features of antennal sensilla in *S. zeamais*.

Sensilla Type	N. (mean ± SE)	Lenght (μm) (mean ± SE)	Diameter (μm) (mean ± SE)	Pores	Wall	Neurons	Tip
*CS1*	17 ± 1	27 ± 1.8	1.2 ± 0.04	Uniporous	Single Thick	6	Round
*CS2*	40 ± 3	12 ± 0.5	0.86 ± 0.04	Aporous	Single Thick	1	Sharp branched
*BS1*	208 ± 13	12 ± 0.4	1 ± 0.03	Multiporous	Single Thin	2–3	Round
*BS2*	102 ± 8	22 ± 1.4	0.9 ± 0.02	Multiporous	Single Thick	2–3	Round
*GPS*	40 ± 7	6.8 ± 0.2	0.83 ± 0.01	Multiporous	Double walled	3–4	Finger-like
